# Family income and young adolescents’ perceived social position: associations with self-esteem and life satisfaction in the UK Millennium Cohort Study

**DOI:** 10.1136/archdischild-2015-309651

**Published:** 2016-03-08

**Authors:** Rienke Bannink, Anna Pearce, Steven Hope

**Affiliations:** 1 Department of Public Health, Erasmus University Medical Center Rotterdam, Rotterdam, The Netherlands; 2 Department of Population, Policy and Practice, UCL Institute of Child Health, London, UK

**Keywords:** Child Psychology, Adolescent Health, Comm Child Health

## Abstract

**Background:**

Self-esteem and life satisfaction are important aspects of positive mental health in young people, and both are socially distributed. However, the majority of evidence is based on socioeconomic characteristics of the family. As children enter adolescence and gain independence, perceptions of their own social position are likely to influence mental health.

**Design and objectives:**

Using data on 11-year-olds from the UK Millennium Cohort Study, we investigated associations of both family income and young adolescents’ perception of their social position with self-esteem and life satisfaction. We hypothesised that there would be differences in the impact of perceived social position on positive mental health when investigating the full scale scoring distribution or the bottom of the distribution. Therefore, we estimated proportional odds for having *greater* positive mental health (across the distribution of scores) and ORs for *poor* outcomes (lowest 10% scores).

**Results:**

The likelihood of *greater* self-esteem and life satisfaction increased with income; similarly, the risk of having *poor* self-esteem and life satisfaction increased as income decreased. Young adolescents who perceived their family as poorer than their friends (instead of about the same) were less likely to have *greater* self-esteem and life satisfaction and were more likely to have *poor* outcomes. Young adolescents who perceived their family as richer were more likely to have *poor* self-esteem, but were not less likely to have *greater* self-esteem. For life satisfaction, young adolescents who perceived their families as richer were less likely to have *greater* and more likely to have *poor* life satisfaction.

**Conclusions:**

Policies to redistribute income in families with children are likely to benefit the mental health of young people. However, it is also important to consider the impact of social comparison on young people's mental health as they enter adolescence.

**What is already known on this subject**

- Socioeconomic inequalities in the mental health of adults and children are widely established.
- Research suggests that adolescents from lower income families have worse outcomes, as may those who perceive their family to be poorer (compared with families of peers).
- However, there is scant research evidence examining the association between perceived social position and positive mental health of young people.

**What this study adds**

- Low family income was associated with lower self-esteem and life satisfaction in a representative sample of UK 11-year-olds.
- After adjustment for income, young people who perceived their family to be poorer (rather than ‘about the same’) than their peers had lower self-esteem and life satisfaction.
- Those who perceived their family to be richer (as opposed to about the same) than their peers also had worse self-esteem and life satisfaction.
- Future research should focus on the pathways from perceived social position to positive mental health outcomes in young adolescents.

## Introduction

Traditionally, research examining child health inequalities has focused on the socioeconomic circumstances of the child's parents and family (such as household income or maternal education).^1–6^ However, as a child starts to gain independence from their family, awareness of their social position relative to peers is likely to increase.^7^ Research suggests that adolescents will have worse health outcomes when they perceive their family to be lower in the socioeconomic hierarchy compared with families of peers.^8^ However, there is scant research evidence examining the association between perceived social position and health (particularly mental health) among children who are within the transitional period between childhood and adolescence (referred to as ‘young adolescents’ hereafter). In addition, there is a paucity of research comparing the social gradients in health according to perceived social position and family socioeconomic circumstances, or differentiating the impact on a young person's mental health of being richer, as well as poorer, than their peers.

Mental health problems are common in adolescence^9^ ^10^ and are a priority for researchers and policy makers alike.^11^ ^12^ Two important aspects of positive mental health are self-esteem (evaluative attitude towards the self^13^) and life satisfaction (a global judgement of one's life^14^). Low self-esteem and low life satisfaction are associated with poorer academic achievement and anxiety, depression and eating pathology.^15^ ^16^ Given the importance of self-esteem and life satisfaction in the development and general functioning of adolescents, insight into the effect of social inequality on self-esteem and life satisfaction is required. However, relatively few researchers have examined inequalities in young people's life satisfaction^17–21^ or self-esteem.^17^ ^20–22^

A comparison of inequalities in positive mental health according to family socioeconomic circumstances and young adolescents’ perceptions of their social position will increase our knowledge of the mechanisms behind socioeconomic inequalities, inform policy and practice and ultimately contribute to global efforts to inequality reduction. This study investigates the associations of family income and young adolescents’ perception of their social position with self-esteem and life satisfaction, using a large, contemporary UK cohort of 11-year-olds born at the turn of the century.

## Methods

### Subjects and design

We used data from the Millennium Cohort Study (MCS), a longitudinal study of children born in the UK between September 2000 and January 2002, which has been described elsewhere.^23^ The first study contact with the cohort child was around age 9 months, when information was collected on 72% of those approached, providing data on 18 818 infants (our analyses were restricted to 18 296 singletons). Survey interviews were carried out by interviewers in the home with the main respondent (usually the mother).The fifth data collection sweep was conducted in 2012–2013, when the children were age 11, and 13 112 (71.7%) main respondents participated. Cohort children were also invited to complete a survey in this sweep, and 12 824 (97.8%) did so. Response weights are used in all analyses to account for sample design and attrition.^24^ ^25^

Data were obtained from the UK Data Archive, University of Essex in March 2014. Ethical approval for the fifth sweep of the MCS was granted by the Northern and Yorkshire multicentre research ethics committee in July 2011 (Ethics Committee reference: 11/YH/0203).^26^ Further information about the MCS can be found elsewhere (http://www.cls.ioe.ac.uk/MCS).

### Measurements

#### Exposures

We examined two socioeconomic measures; one pertaining to the objective socioeconomic circumstances of the household (family income) and one capturing the young adolescents’ subjective perceptions of their family's wealth relative to their peers (perceived social position).

*Family income*. Family income was reported by the main respondent at the 11-year sweep. Income quintiles were derived using a modified Organisation for Economic Cooperation and Development (OECD) equivalence scale, ranging from the lowest income group (first quintile) to the highest income group (fifth quintile). Missing income data were multiply imputed by the Centre for Longitudinal Studies before data from the sweep were deposited.^24^

*Perceived social position*. The cohort children responded to the item, ‘Compared to your friends, is your family *richer*, *about the same, poorer* or *don't know*?’. Of all children, 1.3% (n=172) did not answer this question. Since the focus of the paper was on a perceived rating of comparative social position, those who answered *don't know* were excluded from the main analyses, including this measure. However, these children were included in analyses examining only family income and results focusing on the *don't know* category are provided in online supplementary appendices 1–3.

10.1136/archdischild-2015-309651.supp1 Supplementary appendices

#### Outcomes

Two measures were examined representing different aspects of positive mental health. The majority of children had high scores indicating good mental health. Because the impact of perceived social position across the distribution of mental health may be different from impacts at the extreme of the distribution (ie, very poor mental health), we examined both outcomes as ordinal and dichotomised variables.

*Self-esteem*. Self-esteem was measured with a shortened and adapted version of Rosenberg's Self-Esteem Scale,^13^ comprising five items reflecting a positive view of self (see online supplementary appendix 4). Item responses were reported on a four-point scale ranging from *Strongly disagree* to *Strongly agree*. The items showed good internal consistency (Cronbach's alpha=0.74) and results from principal component analysis (PCA) showed that items loaded on a single construct. Responses were summed to provide a score ranging from 5 to 20, with higher scores indicating *greater* self-esteem. Most children had a high self-esteem score (25th, 50th and 75th centiles: scores 15, 17 and 19, respectively). In addition, children were classified as having *poor* self-esteem if they were in the bottom 10% of scores (score range: 5–14).

*Life satisfaction.* Life satisfaction was measured with six items reflecting children's feelings about different parts of their life, including school, family and friends (see online supplementary appendix 4). Responses were reported on a seven-point scale ranging from *Not at all happy* to *Completely happy*. Internal consistency of the scale was good (Cronbach's alpha=0.83), and PCA results showed that items loaded on a single construct. Responses to items were summed to provide a score ranging from 6 to 42, with higher scores indicating *greater* life satisfaction. Most children had a high life satisfaction score (25th, 50th and 75th centiles: scores 33, 37 and 40, respectively). In addition, children were classified as having *poor* life satisfaction if they were in the bottom 10% of scores (score range: 6–28).

Seven hundred and fifty-two children (6.4%) were missing a self-esteem score and 281 (2.4%) a life satisfaction score, largely due to missing entries for just one or two items on the self-esteem or life satisfaction scale. Therefore, we rescaled the average using the other completed items when children had responded to at least three items of the self-esteem or life satisfaction scale. This reduced missingness to 1.5% for the self-esteem scale and 0.4% for the life satisfaction scale. We ran sensitivity analyses with the self-esteem and life satisfaction scores rescaled when only a single item was missing and for complete data on the scales. Patterns of results were largely similar to those reported (data not shown).

*Potential confounders*. We selected potential confounders that were hypothesised to relate to the outcomes, family income and perceived social position (maternal age at first live birth, child's ethnicity and gender). Furthermore, in the case of the child's perceived social position, analyses adjusted for family income in order to test if the influence of perceived social position persisted after taking into account family socioeconomic circumstances. Missing data on the confounding variables were as follows: ethnicity (n=70; 0.6%) and maternal age at first live birth (n=837; 6.6%).

We examined whether the association between socioeconomic exposures and outcomes varied by gender. No interaction with gender was found for any model, and so all results are presented for girls and boys combined.

We ran sensitivity analyses with maternal education as an additional potential confounder. Patterns of results were largely similar to those reported. Furthermore, results were very similar when maternal education was used as an alternative measure of socioeconomic circumstances in place of family income (not shown).

### Statistical analyses

All analyses were conducted in STATA/SE V.13 (Stata, Texas, USA), using ‘svy’ commands to allow for the sampling design and attrition up to age 11 years. Kendall's tau-b test was used to examine the extent to which family income and perceived social position were associated. Two types of regression analyses were conducted. Proportional ORs (PORs) and 95% CIs were estimated using ordinal regression analysis to examine positive mental health (ordinal scores), according to family income and perceived social position. ORs and 95% CIs were estimated using logistic regression to examine *poor* self-esteem and life satisfaction (binary outcomes), according to family income and perceived social position. Models were estimated before and after adjustment for confounders.

## Results

### Family income and perceived social position

Young adolescents whose families were in higher income quintiles were more likely to rate their family as richer than their peers*;* however, the association was weak (Kendall's tau-b=0.04; p<0.001) (see online supplementary appendix 1), indicating that the two measures capture different aspects of the socioeconomic experience.

### Inequalities in self-esteem

The proportional odds analysis showed that young people from families in lower income quintiles were less likely to have *greater* self-esteem compared with those from the highest income quintile, both before and after adjustment (table 1). Young adolescents who rated their family as poorer were also less likely to have *greater* self-esteem compared with young adolescents who rated their family as about the same (Model A2: POR=0.45, 95% CI 0.37 to 0.56). Young adolescents who considered their families to be richer were similar to young adolescents who perceived their family to be about the same (Model A2: POR=1.09, 95% CI 0.94 to 1.26). These differences remained after adjustment for confounding and family income.

**Table 1:** Associations of family income and young adolescents’ perceived social position with self-esteem (N=11 618)

	*Greater* self-esteem (ordinal scores)		*Poor* self-esteem (bottom 10% scores)*	
	Model A1	Model A2†	Model B1	Model B2†
	Proportional OR (POR) (95% CI)	POR (95% CI)	OR (95% CI)	OR (95% CI)
Family income				
Lowest income quintile	0.81 (0.70 to 0.94)	0.76 (0.64 to 0.89)	1.86 (1.48 to 2.33)	1.70 (1.27 to 2.29)
Second quintile	0.79 (0.70 to 0.89)	0.80 (0.71 to 0.90)	1.64 (1.32 to 2.03)	1.45 (1.12 to 1.87)
Third quintile	0.87 (0.77 to 0.98)	0.89 (0.89 to 1.00)	1.36 (1.07 to 1.73)	1.27 (0.99 to 1.63)
Fourth quintile	0.86 (0.77 to 0.96)	0.88 (0.79 to 0.99)	1.15 (0.93 to 1.43)	1.11 (0.88 to 1.40)
Highest income quintile	Ref.	Ref.	Ref.	Ref.
Compared with your friends, is your family?‡				
Poorer	0.45 (0.36 to 0.56)	0.45 (0.37 to 0.56)	2.76 (2.05 to 3.70)	2.62 (1.95 to 3.53)
About the same	Ref.		Ref.	Ref.
Richer	1.15 (0.99 to 1.33)	1.09 (0.94 to 1.26)	1.25 (0.98 to 1.59)	1.37 (1.07 to 1.75)

*Score range: 5–14 (out of a possible range of 5–20).

†Models A2 and B2 are adjusted for maternal age at first live birth, sex and ethnicity of the child. The analysis of perceived social position also adjusted for family income.

‡N=9785; 1895 young adolescents who answered *don't know* were excluded from these analyses.

The binary odds analysis (focusing on *poor* outcomes) indicated that young adolescents from families in lower income quintiles were more likely to have *poor* self-esteem compared with young adolescents whose family income was in the highest quintile (table 1). Similarly, young adolescents who rated their family as poorer were more likely to have *poor* self-esteem than young adolescents who rated their family as about the same (Model B2: OR=2.62, 95% CI 1.95 to 3.53). However, young adolescents who rated their family as richer were also slightly more likely to have *poor* self-esteem (Model B2: OR=1.37, 95% CI 1.07 to 1.75). The findings were similar before and after adjustment.

### Inequalities in life satisfaction

Young adolescents from lower income quintile families were less likely to report *greater* life satisfaction compared with young adolescents whose family income was classified in the highest income quintile, both before and after adjustment (table 2). Young adolescents who rated their family as richer (Model C2: POR=0.76, 95% CI 0.65 to 0.89), as well as young adolescents who rated their families as poorer (Model C2: POR=0.33, 95% CI 0.27 to 0.39), were less likely to have *greater* life satisfaction (compared with young adolescents who rated their family as about the same). This remained the case after adjustment for confounding and family income.

**Table 2:** Associations of family income and young adolescents’ perceived social position with life satisfaction (N=11 745)

	*Greater* life satisfaction (ordinal scores)		*Poor* life satisfaction (bottom 10% scores)*	
	Model C1	Model C2†	Model D1	Model D2†
	Proportional OR (POR) (95% CI)	POR (95% CI)	OR (95% CI)	OR (95% CI)
Family income				
Lowest income quintile	0.74 (0.64 to 0.87)	0.71 (0.60 to 0.85)	1.81 (1.44 to 2.23)	2.11 (1.58 to 2.83)
Second quintile	0.75 (0.66 to 0.84)	0.75 (0.66 0 0.86)	1.50 (1.20 to 1.87)	1.65 (1.26 to 2.15)
Third quintile	0.88 (0.79 to 0.98)	0.88 (0.78 to 0.98)	1.33 (1.09 to 1.63)	1.43 (1.14 to 1.79)
Fourth quintile	0.92 (0.83 to 1.02)	0.92 (0.83 to 1.02)	0.98 (0.78 to 1.22)	1.01 (0.81 to 1.27)
Highest income quintile	Ref.	Ref.	Ref.	Ref.
Compared with your friends, is your family?‡				
Poorer	0.31 (0.26 to 0.37)	0.33 (0.27 to 0.39)	3.76 (2.87 to 4.92)	3.58 (2.72 to 4.70)
About the same	Ref.		Ref.	Ref.
Richer	0.78 (0.67 to 0.92)	0.76 (0.65 to 0.89)	1.68 (1.34 to 2.12)	1.77 (1.41 to 2.23)

*Score range: 6–28 (out of a possible range of 6–42).

†Models C2 and D2 are adjusted for maternal age at first live birth, sex and ethnicity of the child. The analysis of perceived social position also adjusted for family income.

‡N=9869; 1895 young adolescents who answered *don't know* were excluded from these analyses.

Young adolescents from lower income quintile families were more likely to have a *poor* life satisfaction compared with young adolescents from the highest income quintile families (table 2). Young adolescents who rated their family as poorer (Model D2: OR=3.58, 95% CI 2.72 to 4.70) or richer (Model D2: OR=1.77, 95% CI 1.41 to 2.23) were significantly more likely to have a *poor* life satisfaction. Associations were similar before and after adjustment.

## Discussion

### Summary of findings

This is the first study, to our knowledge, that has compared the associations of family income and perceived social position with positive mental health in young adolescents. We found that while the majority of 11-year-olds had high self-esteem and life satisfaction, levels of both tended to decline as family income decreased. In addition, the risk of having *poor* self-esteem and life satisfaction increased as income fell. Young adolescents who perceived their family as poorer were less likely to have *greater* self-esteem and life satisfaction and more likely to fall in the bottom 10% of scores. Patterns of positive mental health for young adolescents who perceived their families to be richer (as opposed to about the same) were more complex. For self-esteem, differences were only observed for the lowest scores: young adolescents who perceived themselves to be richer were slightly more likely to have *poor* self-esteem, whereas there were no differences in self-esteem when looking at *greater* self-esteem across the distribution. For life satisfaction, the findings were consistent across the distribution: young adolescents who perceived their families to be richer were less likely to have *greater* scores and more likely to have *poor* life satisfaction.

### Comparison with other findings

There is a large body of research examining socioeconomic inequalities in the health of children.^1–6^ However, the existence of health inequalities within the transitional period from childhood to adolescence has not yet been well researched. This has possibly been exacerbated by difficulties in obtaining an accurate picture of family socioeconomic circumstances directly from the young person (in studies where they are the sole respondent).^27^ Furthermore, it is unclear whether parental socioeconomic circumstances are an appropriate measures of a child's social position.^27^ For example, some research has suggested that differences in health by family socioeconomic circumstances, evident in childhood and adulthood, are lessened in adolescence because of the increased influence of peers, school environment and youth culture.^7^ However, in the current study, we found that, at age 11, the socioeconomic situation of the family (household income) was still influential. Importantly, we also found that in children as young as 11, perceptions of their position in the socioeconomic hierarchy can have a negative impact on their mental health, even after controlling for family income. This indicates that these measures represent different aspects of the socioeconomic experience (supported by the weak association found between family income and social position in this study and in earlier work^17^ ^28–30^). In particular, we found that those who perceived themselves as being richer (as well as poorer) had worse outcomes. Studies in older adolescents^17–22^ have shown that those who perceived their family as poorer compared with their friends’ families had lower self-esteem and life satisfaction. However, perceived social position was analysed in a way that did not always allow for the possibility that young people who perceived themselves as richer than their peers might also have worse outcomes. Our findings indicate that in doing so they may have overlooked important associations.

### Strengths and limitations

This study has examined inequalities in different positive mental health scales, using a large, contemporary and nationally representative cohort of early adolescents in the UK. The results varied according to the two socioeconomic measures, which although correlated were intended to capture different aspects of the child's socioeconomic circumstances. The child's perceived social position was subjective and with reference to the child's own social circles, whereas income captured the family financial circumstances (compared with families with children of a similar age across the UK). Although life satisfaction and self-esteem were not assessed using standard measures, the scales showed good internal consistency and items loaded on a single construct. Furthermore, life satisfaction was a multi-item scale, which is considered to be preferable to single-item scales that are often used to measure this outcome.^31^ Correlations of self-esteem and life satisfaction with a validated tool for assessing children's mental health status by parents and teachers (the Strengths and Difficulties Questionnaire) were examined and the directions of the associations were as expected (data available on request). Weights were used in all analyses to account for the sampling design and differential response to age 11 years. These weights cannot account for item missingness, although our analytic sample consisted of the majority (91.6%) of MCS children who took part in the 11-year sweep.

### Implications for policy and research

The mental health of young people is critical for educational attainment, employment and adult health. We have shown substantial inequalities in self-esteem and life satisfaction in early adolescence, which may persist into adult life. In addition, we found that the influence of perceived social position on positive mental health was independent of household income, and perceptions of being different from peers (richer or poorer) appeared detrimental to mental health. This implies that there may be multiple mechanisms through which socioeconomic circumstances influence the mental health of a young person, related both to absolute disadvantage (such as not being able to afford basic necessities) and the psychosocial consequences of disadvantage (where feeling different from peers may in itself have negative consequences).

Policies to redistribute income are likely to benefit the mental health of young people from poorer families through the reduction of material disadvantage. However, the impact of inequalities on mental health may additionally involve more subtle psychosocial factors, which could be detrimental for *all* young people living in unequal societies, such as the UK. While income redistribution has the potential to benefit everyone, an exploration of the impact of pressures created by social comparison at the level of the individual is also necessary. This will help to inform health-based and school-based interventions to alleviate or prevent poor mental health. Health professionals have an important role to play in health inequality reduction, through considering the root causes of patients’ mental health, and (in the case of children) taking a family-centred approach.^32^ For young people, it is essential to also consider wider influences, including neighbourhoods, schools and peers.
