# Supplementary material for: Family income and young adolescents’ perceived social position: associations with self-esteem and life satisfaction in the UK Millennium Cohort Study
Source: Arch Dis Child. 2016 Mar 8;101(10):917–21. doi: 10.1136/archdischild-2015-309651 (PMC5050283; doi:10.1136/archdischild-2015-309651)
Supplement: Supplementary appendices [file archdischild-2015-309651supp.pdf]

Appendix 1. Relationship between family income and young adolescents' perceived social position[a] (N = 11793)

	**Compared to your friends, is your family?**			
	Poorer	About the same	Richer	Don't know
**Family income**	% (N)	% (N)	% (N)	% (N)
Lowest income quintile	21.1 (105)	19.2 (1542)	17.0 (160)	22.4 (413)
Second quintile	29.5(120)	20.2 (1690)	17.6 (174)	21.3 (403)
Third quintile	19.3 (95)	20.4 (1849)	19.8 (193)	18.9 (372)
Fourth quintile	18.2 (88)	20.4 (1777)	19.5 (191)	19.3 (379)
Highest income quintile	11.9 (61)	19.9 (1623)	26.2 (230)	18.2 (328)
**Total**	100 (469)	100 (8481)	100 (948)	100 (1895)

[a] Kendall's tau-b: 0.04 (p <0.001): *don't know* group excluded from this analysis.

Appendix 2. Associations of young adolescents' perceived social position (i.e. *don't know* versus *about the same*) with self-esteem (N = 10229)

	***Greater* self-esteem (ordinal scores)**		***Poor* self-esteem (bottom 10% scores)[a]**	
	Model A1	Model A2[b]	Model B1	Model B2[b]
	Proportional OR (95% CI)	Proportional OR (95% CI)	OR (95% CI)	OR (95% CI
**Compared to your friends, is your family?**				
About the same	Ref.	Ref.	Ref.	Ref.
Don't know	0.74 (0.66 – 0.82)	0.72 (0.66 – 0.81)	1.41 (1.27 – 1.56)	1.40 (1.27 – 1.56)

OR = Odds ratio.

[a] Score range: 5 – 14 (out of a possible range of 5 – 20).

[b] Model A2 and B2 are adjusted for maternal age at first live birth, sex and ethnicity of the child. Analysis of perceived social position also adjusted for family income.

Appendix 3. Associations of young adolescents' perceived social position (i.e. *don't know* versus *about the same*) with life satisfaction (N = 10335)

	***Greater* life satisfaction (ordinal scores)**		***Poor* life satisfaction (bottom 10% scores)[a]**	
	Model C1	Model C2[b]	Model D1	Model D2[b]
	Proportional OR (95% CI)	Proportional OR (95% CI)	OR (95% CI)	OR (95% CI
**Compared to your friends, is your family?**				
About the same	Ref.	Ref.	Ref.	Ref.
Don't know	0.71 (0.64 – 0.79)	0.71 (0.64 – 0.79)	1.74 (1.46 – 2.07)	1.73 (1.46 – 2.06)

OR = Odds ratio.
[a] Score range: 6 – 28 (out of a possible range of 6 – 42).
[b] Model C2 and D2 are adjusted for maternal age at first live birth, sex and ethnicity of the child. Analysis of perceived social position also adjusted for family income.

Appendix 4. Self-esteem and life satisfaction scale items

**Self-esteem scale**	**Life satisfaction scale**
On the whole, I am satisfied with myself	How do you feel about your school work?
I feel that I have a number of good qualities	How do you feel about the way you look?
I am able to do things as well as most other people	How do you feel about your family?
I am a person of value	How do you feel about your friends?
I feel good about myself	How do you feel about the school you go to?
	How do you feel about your life as a whole?
